# Functional Metabolomics Analysis Elucidating the Metabolic Biomarker and Key Pathway Change Associated With the Chronic Glomerulonephritis and Revealing Action Mechanism of Rhein

**DOI:** 10.3389/fphar.2020.554783

**Published:** 2020-09-25

**Authors:** Wei Yu, Wei Yang, Ming-Yan Zhao, Xiang-Lin Meng

**Affiliations:** Department of Intensive Care Unit, First Affiliated Hospital of Harbin Medical University, Harbin, China

**Keywords:** metabolomics, therapeutic mechanism, pathway, biomarkers, metabolic pathway, metabolomic analyses

## Abstract

Chronic glomerulonephritis (CGN) as the culprit of kidney failure can increase the mortality of critically ill patients and seriously threatens people’s health all over the world. This study using metabolomics strategy is to reveal the potential therapeutic mechanism-related targets to evaluate the effects of rhein (RH) on CGN rats. Changes of serum metabolites and pathways were analyzed by non-targeted metabolomic method based on liquid chromatography-mass spectrometry (LC-MS) combined with ingenuity pathway analysis. In addition, the levels of biochemical indicators were also detected. A total of 25 potential biomarkers were identified to express serum metabolic turbulence in CGN animal model, and then 16 biomarkers were regulated by RH trending to the normal states. From metabolite enrichment and pathway analysis, pharmacological activity of RH on CGN were mainly involved in six vital metabolic pathways including phenylalanine, tyrosine and tryptophan biosynthesis, phenylalanine metabolism, arachidonic acid metabolism, tricarboxylic acid cycle (TCA cycle), alanine, aspartate, and glutamate metabolism, arginine and proline metabolism. It suggested CGN treatment with RH, which may be mediated *via* interference with metabolic pathway such as amino acid metabolism, arachidonic acid metabolism, and TCA cycle to regulating inflammation, oxidation response and immune regulation against CGN. It showed that metabolomics method offer deeply insight into the therapeutic mechanisms of natural product.

## Introduction

CGN as a kind of auto-immunologically mediated glomerular injuries in chronic kidney disease is characterized by circulating inflammatory cells infiltration, glomerular cells proliferation, and extracellular matrix (ECM) accumulation (Trivedi et al., 2017; Bhalla et al., 2019; Liu et al., 2019; Nanda et al., 2019). The pathogenesis of CGN is mainly that the immune complexes activate the complement system leading to the release of cytokines by neutrophils and lymphocytes, eventually causing glomerular damage (Lien and Lai, 2011; Ciuntu, 2016). It is also the most common cause of glomerulosclerosis and end-stage renal disease, which often leads to the patients to appear proteinuria, hematuria, and brings out notable societal and economic burdens on national health systems (Lim et al., 2018). Currently, the clinical treatment of CGN commonly uses antihypertensive, anticoagulant, and hormone and cytotoxic drugs such as corticosteroids, immune-suppressors, angiotensin-converting enzyme inhibitors, angiotensin II receptor blockers, and cyclophosphamide to relieve severe clinical symptoms and kidney failure, which possess insufficient efficacy, high cost, and unpredictable side effects (Okada et al., 2006; Guo et al., 2008; Santoro et al., 2012; Joy et al., 2012; Gao et al., 2019). With the continuously growing incidence, seeking accurate diagnosis technology in early stage and ideal treatment is urgently needed.

Natural products have become a major resource for looking for potential therapeutic candidates by the global research community’s attention. Rhubarbas, one of popular traditional Chinese medicine, have been applied to the treatment of renal diseases and infectious inflammation for a long time (Zhang et al., 2019). Rhein also named 4, 5-dihydroxyanthraquinone-2-carboxylic acid is an active lipophilic anthraquinone compound extracted and separated from rhubarb (Keser et al., 2019). A systematic review and meta-analysis has reported that rhein has beneficial effects on animal models of diabetic nephropathy mediated by ameliorating levels of transforming growth factor-β (TGF-β1), renal fibrosis, metabolism, and oxidative stress status (Hu et al., 2019). RH protected intestinal epithelial-6 (IEC-6) cell against oxidative damage partly *via* PI3K/Akt and Nrf2/HO-1 pathways (Zhuang et al., 2019). RH derivative 4F as a novel anthraquinone compound with better anti-tumor activity through the more stably binding to Rac1 and inhibiting Rac1 promoter activity in cells and down-regulating Rac1 protein expression (Li X. et al., 2020). RH enhanced the number and water content of fecal pellets coupled with mast cells accumulation, increasing the content of interferon (IFN) -γ and decreased the levels of interleukin (IL) -10 in the rat colon (Wu et al., 2019). RH can protect myocardial H9c2 cells against ischemia reperfusion (I/R)-induced apoptosis involved in AKT/GSK3β/p38 pathway, which increased the phosphorylation of AKT and GSK3β, and lowed the p-P38 level (Liu J. et al., 2018). In acute promyelocytic leukemia (APL) cells, RH potentiated all-trans retinoic acid (ATRA)-induced macrophage differentiation in NB4 cells by inducing changes in morphology, expression of the differentiation markers CD11b and CD14, reactive oxygen species (ROS) production, phagocytic activity, and expression of CCR1 and CCR2. In addition, it also induced APL cell death by activating apoptosis and suppressing the mTOR pathway (Heo et al., 2018). Modern pharmacology studies have showed that RH could obviously restrains the proliferation of glomerular mesangial cells and hypertrophy of glomerulus, the production and accumulation of extracellular matrix. In addition, it suppresses mRNA transcription, thrombospondin-1 (TSP-1), and transforming growth factor-beta1 (TGF-β1) expression in renal tubular epithelial cells in order to reduce urinary protein and renal fibrosis, ameliorate renal function to protect against CGN deterioration mediated by regulating MAPK signaling pathway, PI3K-AKT signaling pathway, TGF-β signaling pathway, Wnt signaling pathway, VEGF signaling pathway, and others (Zheng et al., 2008; He et al., 2011; Zeng et al., 2014; Lian et al., 2014; Lin et al., 2017; Wang et al., 2018). It is easy to see that RH protects against CGN in multi-target and multi-level way. However, the molecule mechanisms of RH protecting against CGN on omics level are still unclear.

Metabolomics is an efficient technique in systems biology approach to explore the biochemical phenotype of metabolic disturbations in biofluids and organism caused by disorders and stimulations, which the strategy comes down to metabolic profiling monitored by nuclear magnetic resonance, high-performance liquid chromatography/mass spectrometry, and gas chromatography/mass spectrometry combined with chemometrics analysis to measure conventional biochemical and pathological changes (Zhang et al., 2012a; Zhang et al., 2013; Liang et al., 2014; Zhang A. H. et al., 2014; Li et al., 2016; Liang et al., 2016a; Liang et al., 2016b; Zhang A. et al., 2016; Zhang A. H. et al., 2018; Li et al., 2018). At present, metabolomics has been widely used in the diagnosis, staging, treatment, and prognosis of diseases (Liang et al., 2015a; Liang et al., 2017; Kalantari et al., 2017; Li A. P. et al., 2020; Bussalino et al., 2020). In this study, the therapeutic mechanism of RH on the cationized calf serum albumin (C-BSA) induced-CNG model was investigated by functional metabolomics using LC-MS coupled with ingenuity pathway analysis to discover metabolic biomarkers and pathways changes. It provides novel mechanisms understanding of RH against CGN and basis pharmacological evidence for clinical applications.

## Materials and Methods

### Materials

UPLC grade acetonitrile (ACN) and formic acid (FA) was purchased from Fisher Scientific Corporation (Loughborough, East Midlands, UK); UPLC grade methanol (MeOH) was available from Merck (Darmstadt, Hessen, Germany). Distilled water for the solutions and mobile phase preparation was gained from Wahaha Group Co., Ltd. (Hangzhou, China). Standard substance leucine enkephalin with the purity more than 99.2% and RH with the purity more than 98.7% were respectively obtained from Lonza (Barcelona, Estado Anzoátegui, Spain) and Shanghai Chemical Reagent Co. (Shanghai, China). The chromatogram of RH was detected using HPLC (**Supplementary Figure 1**). Cationized calf serum albumin (C-BSA) were available from Stefan Biosciences Company (Beijing, China). Prednisone Acetate Tablets were obtained from Hisun Pfizer Pharmaceutical Co., Ltd. (Shanghai, China). Pentobarbital sodium and physiologic saline solution were purchased from Tong Ren Tang Chinese Medicine Co., Ltd. (Beijing, China). Ten percent neutral formalin solution was bought from Shanghai Chemical Reagent Company (Shanghai, China). Immunohistochemical kit CD3, CD4, and CD8 monoclonal antibodies were bought from Shanghai Yanhui Biotechnology Co., LTD. (Shanghai, China). The kit of glutathione (GSH), superoxide dismutase (SOD), malonyldialdehyde (MDA) were obtained from Sigma-Aldrich, Inc. (Loughborough, East Midlands, USA). The kit of 24 h urinary protein (24h-UP), serum creatinine (SCr), urea nitrogen (BUN), tumor necrosis factor-α (TNF-α), interleukin-6 (IL-6) were acquired from Roche Applied Science (Basel, Basel-Stadt, Switzerland). Other reagents and chemicals not mentioned in the stage of experiment were of analytical or chromatographic grade commercially available.

### Animals Feeding and Model Establishment

Specified Pathogen Free (SPF) SD healthy male rats weighing 200 ± 20g in 8 weeks old were raised in laboratory animal center where indoor temperature was set at 22 ± 2°C and relative humidity was controlled at 50 ± 3% with 12 h light-dark cycle for 7 days adaptation. The experimental agreement ratified by the Animal Care and Use Committee of **Harbin Medical University** in this study. After 1 week, 32 rats with symmetrical mental state and body weight were randomly divided into four groups (n = 8, each group): control group (Control), model group (Model), prednisone acetate tablets positive group (PA), and RH group (RH). The rats in model, PA, and RH group were injected with 2.5 ml C-BSA solution at bilateral armpits and groin, and then injected again 2.0 ml in the same way at the second week. The tail vein of animals were injected with 0.5 ml C-BSA solution at the third week, then was injected once every other day for a continuous period of 3 weeks. The control group was disposed by saline solution at the same day and place in the same way (Adler et al., 1983; Zhong et al., 2008; Wu et al., 2008).

### Treatment

From the first day of animals modeling, rats in PA group was administered orally prednisone acetate at a dose of 8 mg/kg per day, and the RH group were received 9 mg/ml RH solution *via* intragastric administration one time daily. The control and model group were intragastrically administered with distilled water daily for 6 weeks.

### Biochemical Items Detection

#### Urinary Protein

The 24-h urine of rats in each group from 8:00 pm to 8:00 am was collected before and after treatment, and was measured by the Coomassie G-250 method, which the calculation formula of urinary protein content for 24 h is as follows: total quantity of urine protein for 24 h = urine protein concentration × urine output volume for 24 h.

#### Blood Lymphocyte Subsets

Orbital venous blood sample were collected before and after 12 h RH treatment. One hundred μl blood samples were respectively mixed with 10 μl of CD3+, CD4+, CD8+ monoclonal antibodies and 2 ml hemolysin, and then incubated at room temperature for 15 min in the dark. The mixture was centrifugated at 1,000 r/min, 4°C for 5 min and then gained supernatant was discarded. The residual liquid was added 2 ml PBS to resuspend the pellet, centrifuged at 1,000 r/min for 5 min. The supernatant was discarded again and added 2 ml resuspended cells for peripheral blood lymphocyte subsets analysis.

#### Indicators of Inflammatory Reaction and Oxidative Damage

After the rats were anesthetized, 8 ml blood sample was taken from the abdominal aorta, and then was allowed to stand at room temperature for 30 min until the serum was stratified. Subsequently, the sample was centrifuged at 3,000 r/min for 10 min at 4°C. The upper serum was used to detect the content SCr, BUN, TNF-α, IL-6, GSH, SOD, and MDA according to the ELISA kit instructions.

#### TNF-α and IL-6 mRNA in Kidney Tissue

The 0.1 g kidney tissue from each group was added 1 ml of pre-chilled Trizol for homogenization. The suspension was transferred to 1.5 ml EP tubes resting at room temperature for 5 min, and added 0.2 ml of chloroform, shaken vigorously for 15 s, and stand at room temperature for 2 min. The mixture was centrifuged at 12,000 r/min for 15 min at 4°C. The gained water phase was transferred into another EP tube, added respectively equal volume of isopropanol, 75% ice ethanol, and DEPC water solution to measure the OD value and estimate the purity of RNA that the value from 1.8 to 2.0 is used for reverse transcription. Ten μl of the cDNA obtained from the reverse transcription reaction system were stored in the refrigerator at −80°C until use.

### LC-MS Experiments

#### Sample Processing

Before LC-MS analysis, blood sample collected from abdominal aorta in each group were placed 12 h under 4°C condition. Then, 1.0 ml sample was shifted into 5 ml centrifuge tube and mixed by chromatographically pure methanol in a ratio of 1:4. The mixture was vortexed for 60 s and centrifuged at 12,000 rpm for 12 min. One ml of supernatant was perked through the 0.22 μm filter membrane and push into the instrument for testing within several hours.

Ten μl of each rat serum sample were added to 3,000 μl of the working solution to obtain a QC sample for ratifying and optimizing the chromatographic and MS condition. Ten characteristic peaks were selected for method validation assisted with dynamic background subtraction. For the successive operation of six replicates from the same QC samples, the relative standard deviation % (RSD%) calculation result of Rt and peak areas were respectively 0.79 and 2.10%, and those results were respectively 0.96 and 2.43% for six parallel samples in the repeatability evaluation.

#### Instrument Analysis

The chromatographic analytical procedures were employed on a Waters ACQUITY UPLC system equipped with binary pump, online degasser, auto plate-sampler, thermostatically controlled column compartment, and the analyte was separated by a Waters Acquity UPLC BEH C18 column (2.1 mm × 100 mm, 1.7 μm) combined with a VanGuard Pre-Column precolumn (2.1 mm × 5 mm, 1.7 μm), which the column temperature was set at 30°C. The binary gradient mobile phase system contains mobile phase A (water—0.1% formic acid) and mobile phase B (acetonitrile—0.1% formic acid), and the optimized procedure is described as follows: 0 to 1 min, 2 to 10% B; 1 to 2 min, 10 to 30% B; 2 to 7 min, 30∼50% B; 7∼9 min, 50∼80% B; 9∼10 min, 80∼100% B; 10∼11 min, 100∼100% B; 11∼13 min, 100∼2% B. The flow rate was 0.3 ml/min and the injection volume was 4 μl. The autosampler temperature was set 4°C.

Analytes were straightly imported into a Waters Micromass QTOF microt Synapt High Definition Mass Spectrometer (Manchester, UK) with an electrospray ion source (EIS) containing positive and negative ion mode. The main parameters were set as follows: ion source temperature is 600°C; in positive mode, source voltages was set 5,500 V, de-clustered voltage (DP) is set 80 V and collision energy (CE) is set 40 eV; in negative mode, source voltages are set 4,500 V, DP is set 60 V, and CE is set 30 eV. The MS existing 10 ppm mass errors possess a scan range of m/z (mass/charge ratio) 80–1,500 Da in centroid mode. As a reference to ensure accuracy and reproducibility of instrument, leucine enkaphalin in 0.2 ng/ml was calculated in [M+H]^+^ (556.2771) and [M - H]^-^ (554.2614).

### Statistical Analysis

The primitive MS spectra data were imported into Mass Hunter software in order to change into common data format, and then Progenisis QI 2.0 were applied to carry out noise filtering, peak detection, removal of isotope masses, and alignment of retention time (Rt) and mass (m/z). For refraining from the influence of concentration difference among samples, every ion intensity of each spectrum were normalized, and build a data matrix embodying the information of Rt, m/z value, and the normalized peak area. Multivariate analysis such as unsupervised principal component analysis (PCA), supervised orthogonal partial least squares discriminant analysis (OPLS-DA), S-score plot and variance importance for projection (VIP)-plot was performed in SIMCA-P14.1 software (Umetrics, Sweden). The differential metabolites were selected by the criterion that VIP value ≥1.00 in VIP plot and p <0.05 in independent-sample t-test, and were identified by m/z, Rt, fragmentation patterns coupled with online databases such as HMDB, KEGG, Chemspider as well as LIPIDMAPS. Then, they are verified by the corresponding commercially available reference standards. MetaboAnalyst 4.0 (https://www.metaboanalyst.ca/) is used to establish interaction and pathway analysis based on differential metabolite to highlight the efficacy of RH on CGN rats. Statistical analysis was counted in the student’s t-test.

## Results

### Biochemical Analysis

**Figure 1:**
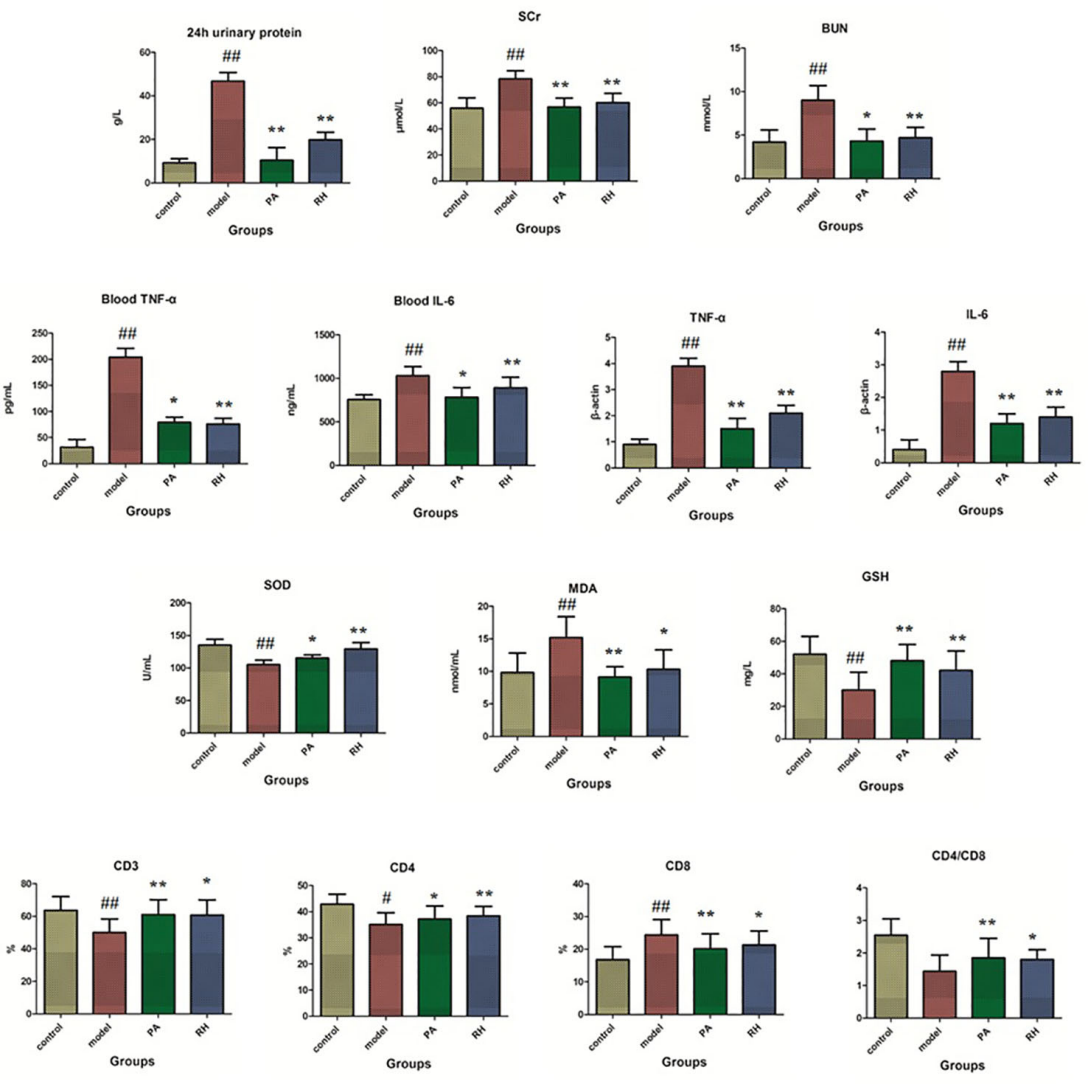
Biochemical detection analysis in rats from control, model, PA, and RH groups. Compared with control group: ^“#”^, p < 0.05; ^“##”^, p < 0.01. Compared with model group: “*”, p < 0.05; “**”, p < 0.01.

As shown in **Figure 1**, 24-h UP quantification as one of the indispensable tests for patients with kidney disease is was measured by collecting the entire urine for 24 h. Renal diseases and physiological conditions such as strenuous exercise can significantly increase urine protein content. Scr is a better indicator of glomerular filtration rate. BUN is a nitrogen-containing compound other than protein in plasma. It is filtered from the glomerulus and excreted from the body. When renal insufficiency is decompensated, BUN will increase. Compared with the control group, the 24-h UP, BUN, and Scr levels in the model group were significantly increased (P < 0.05); compared with the model group, the content of 24-h UP, BUN, and Scr levels were decreased after RH treatment, which the 24-h UP and Scr level possess significantly difference (P < 0.01). MDA, SOD, and GSH are the gold indicators of anti-oxidation performance. Compared with the control group, the serum GSH content and SOD activity in CGN model rats were significantly reduced (P < 0.05), while the MDA content was significantly increased (P < 0.05). After drug intervention, the PA group and RH group can significantly up-regulate the content of GSH in serum, the activity of SOD, and down-regulate the level of MDA, indicating that RH can improve the antioxidant capacity of CGN model rats.

TNF-α is an important inflammatory factor, mainly produced by activated monocytes and macrophages. With the help of the body’s immune function, it has direct and indirect dual anti-cancer effects on the same tumor, causing hemorrhage and necrosis of nutritional blood vessels, inducing IL-2 and IL-6 production, promoting the activity of coagulation factors and tissue factors, and participating in the pathological damage of certain autoimmune diseases (Phan-Lai et al., 2016; McGregor et al., 2019). IL-6 as a lymphokine produced by activated T cells and fibroblasts can make B cell precursors into antibody-producing cells for promoting the growth and differentiation of primitive bone marrow-derived cells and enhancing the lysogeny function of natural killer cells (Zhang A. et al., 2014; Zhou et al., 2016). Compared with the control group, the levels of TNF-α and IL-6 in peripheral blood were significantly increased in the model group (P < 0.05). After RH treatment, TNF-α content was reduced (P < 0.05) and IL-6 content was significantly reduced compared with model rats (P < 0.01). The levels of TNF-α and IL-6 mRNA changes in kidney tissues has the same trend (P < 0.05).

T lymphocytes are the most important cell group in the body’s immune system. When the number and function of different lymphocyte subgroups are abnormal, it can lead to immune disorders and a series of pathological changes (Zajonc and Flajnik, 2016). CD3 molecules can be expressed on the surface of mature T lymphocytes, but CD4 and CD8 cannot be expressed on the surface of mature T lymphocytes at the same time (Carvalho et al., 2015; Clénet et al., 2017). Therefore, mature T lymphocytes can be divided into two subgroups of CD4 T cells and CD8 T cells. CD3 lymphocyte subgroup analysis is an important indicator for detecting cellular and humoral immune functions, and it generally reflects the current immune function, state and balance level of the body (Kim et al., 2018). The CD4/CD8 more than 2.5 indicates that the cellular immune function is in an “overactive” state and is prone to appear autoimmune reactions such as rheumatoid arthritis as well as type I diabetes. Compared with the control group, the level of peripheral blood CD3, CD4, and CD4/CD8 ratios of the model group were notably decreased, and CD8 content was significantly increased (P < 0.05). After RH treatment, the content CD3, CD4, and CD4/CD8 ratios in peripheral blood were significantly increased (P < 0.05), and CD8 level was importantly reduced (P < 0.01).

### Metabolic Biomarkers Changes After RH Treatment

**Figure 2:**
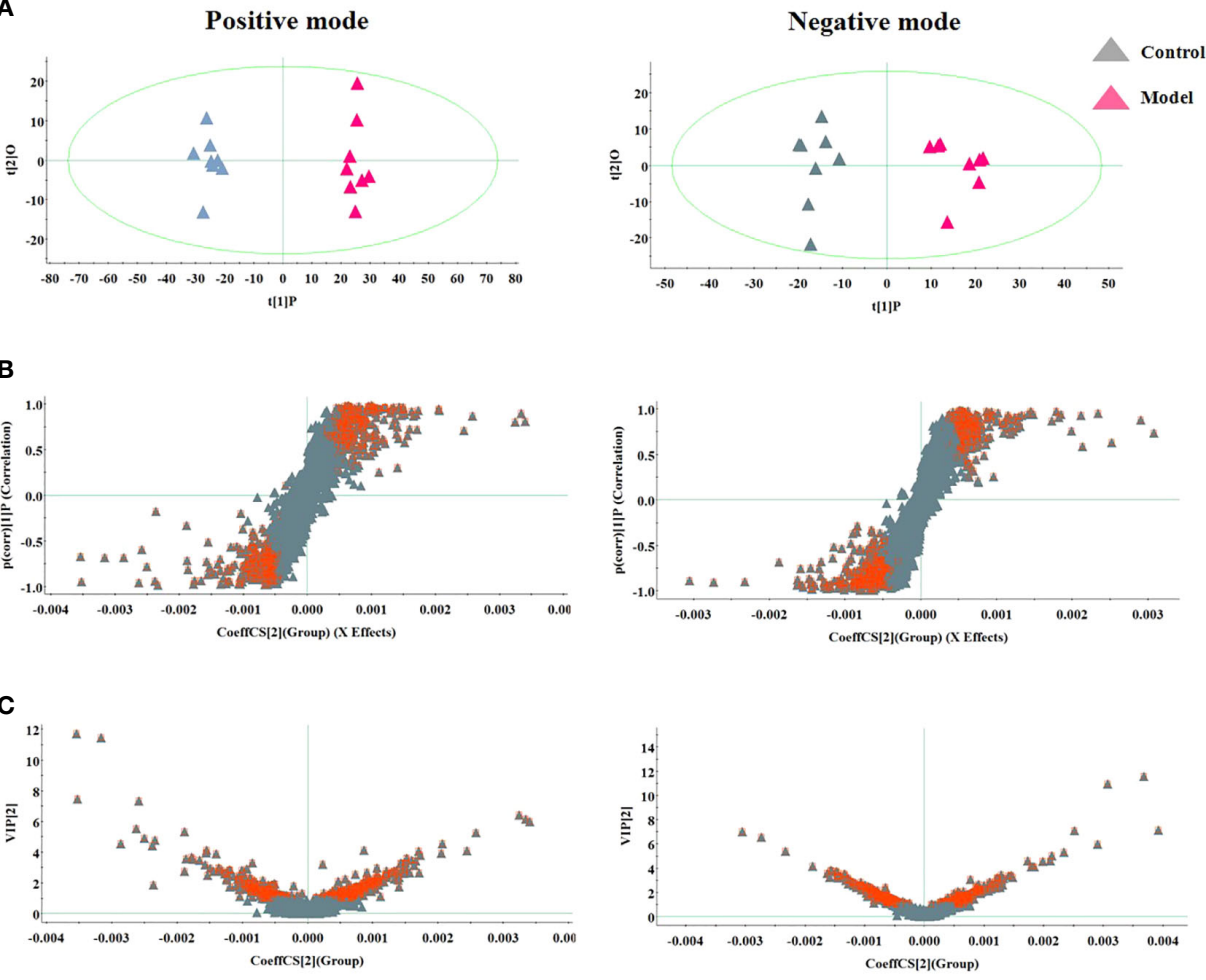
Multivariate analysis of serum metabolites in rats between control and model group for selecting differential metabolites to exploring the pathogenesis, including **(A)** OPLS-DA score plot, **(B)** S-plot of OPLS-DA model, and **(C)** VIP-plot of OPLS-DA model.

**Figure 3:**
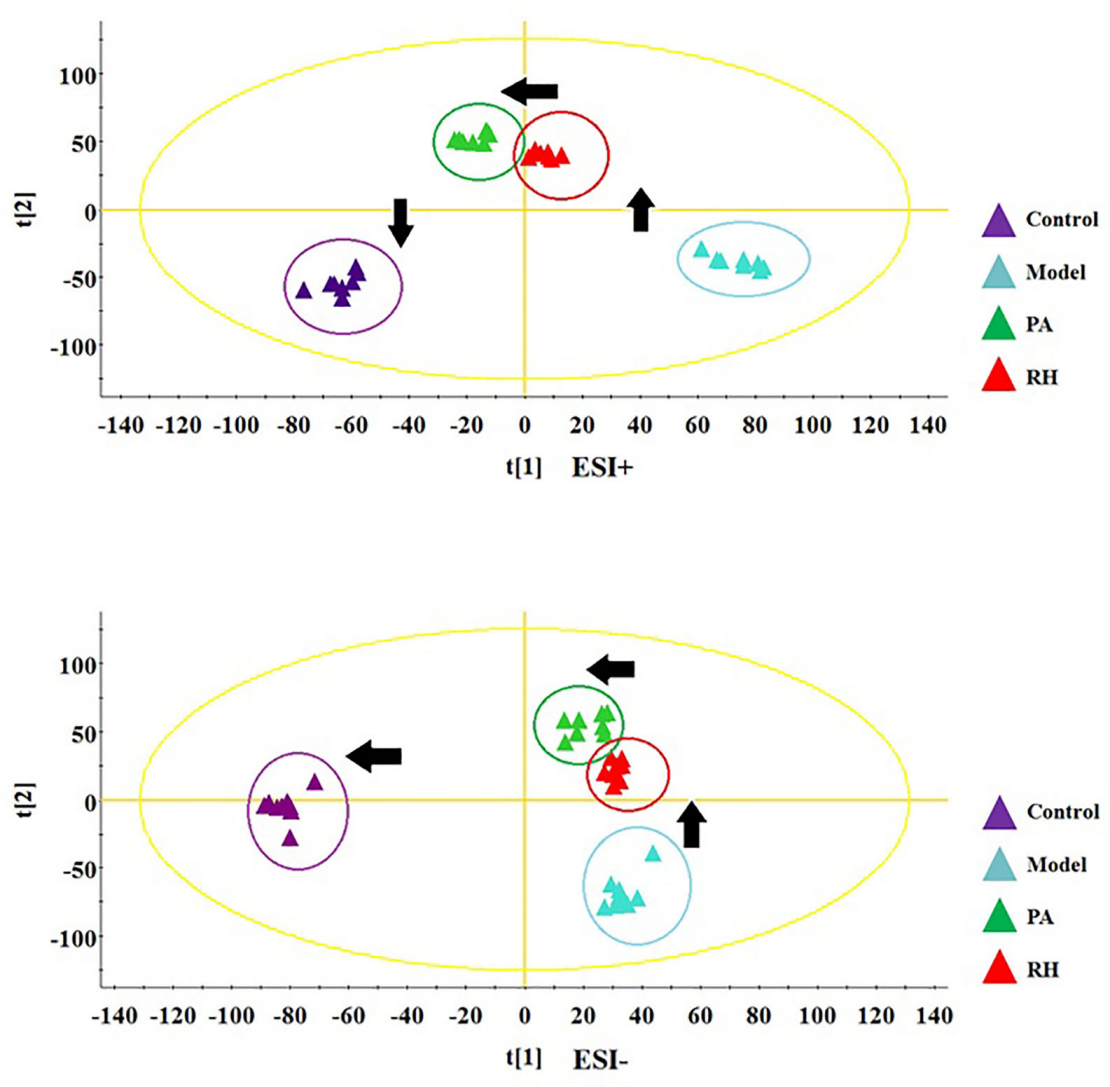
PCA score plot track of serum metabolites changes in rats in the control, model, PA, and RH group in positive ion mode (up) and negative ion mode (down).

During the LC-MS detection, total ion chromatograms of plasma present better behavior including good peak shape, temperate intensity, and clearly separation and indicating that gradient elution procedure in this study is appropriate. But, LC-MS spectra displayed no clear discrimination between each group on account of the complication of the spectra. In order to enlarge the separation and identify the metabolites for model evaluation, a supervised OPLS-DA discriminant analysis approach was applied to highlight the variation between the healthy and CGN model rats. The model evaluation parameter R2X, R2Y, and Q2 values were more than 0.357, 0.982, and 0.918, which implies that the models were accuracy and had predictive abilities. As shown in **Figure 2A**, there was a clearer separation between control group and model group in either the positive or negative modes indicating that CGN model establishment is successfully resulting to significant metabolic change in the rats. S-plot, VIP plot dated from OPLS-DA models and t-test were employed to further filter the metabolites associated with pathology of CGN, which are shown in **Figures 2B, C**. With VIP values above 1.0 and p-values less than 0.05, 25 metabolites in the plasma including isocitric acid, ornithine, 5’-methylthioadenosine, 3-hydroxyanthranilic acid, citric acid, argininosuccinicacid, uric acid, asparagine, tryptophan, glutamine, SM(d18:1/22:0), cervonoylethanolamid, cysteinylglycine, hydroxytyrosol, cyclic GMP, prostaglandin F2a, taurocholic acid, pyruvic acid, phenylalanine, arachidonic acid, LysoPC(17:0), LysoPC(15:0), palmitoleic acid, salbutamol, PE(15:0/20:1) were deemed as potential metabolites that were correlated with CNG in molecule level, and the basic information such as molecular formula, compound name, corresponding m/z, VIP value was listed in **Table S1**. After the CGN treatment, unsupervised PCA was performed on all groups in the study as shown in **Figure 3**. In the positive and negative modes, the samples from the four different groups showed clear separation, and it is easy to see that the clearer separation exist between control group model group than PA and RH group, PA is closer trend to control group than RH group.

**Figure 4:**
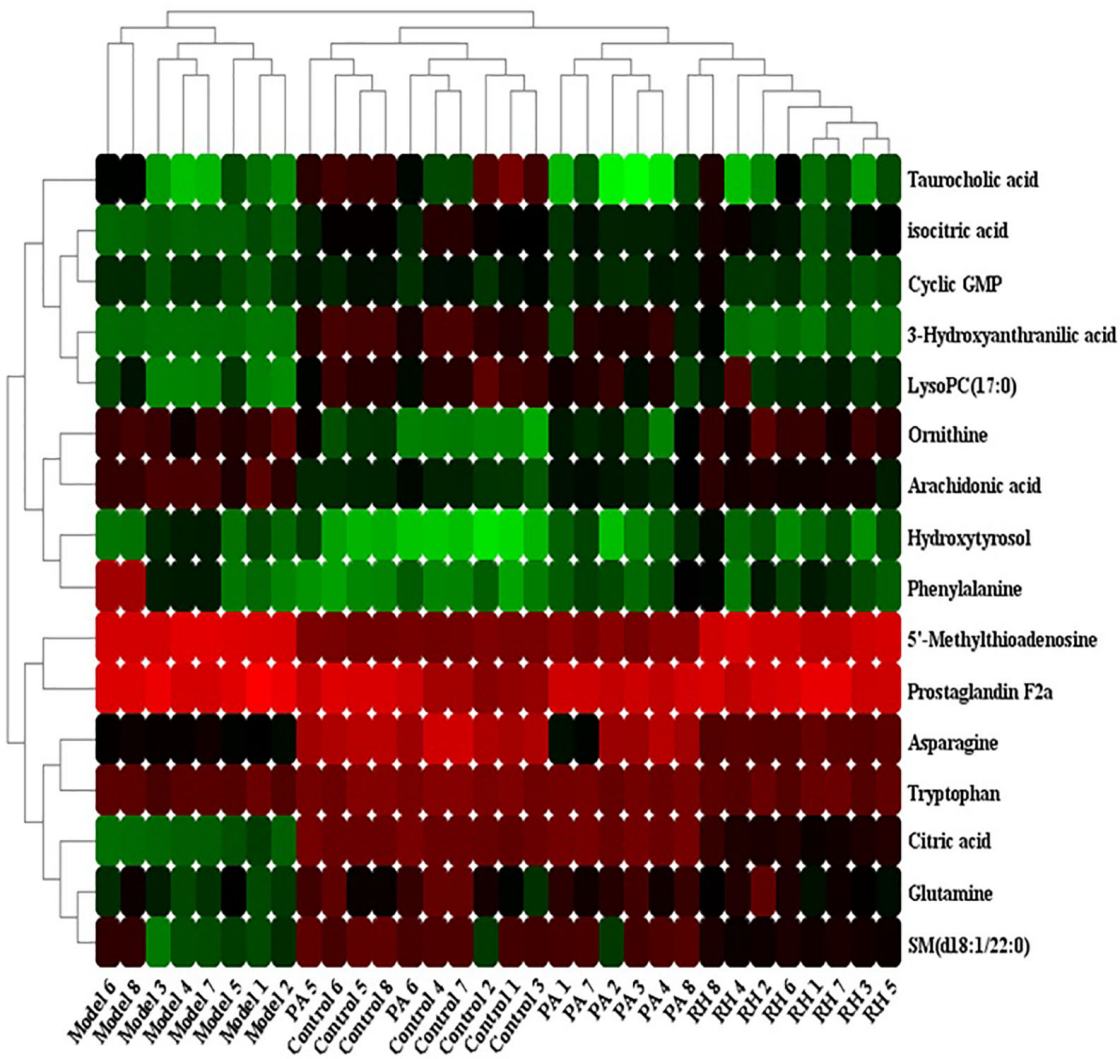
Heatmap visualization for significant changes in 16 potential biomarker candidates between control, model, PA, and RH group. Note: Red color indicates increased metabolite content, green color indicates reduced metabolite content.

The relative peak area was used as the evaluation index. Comparing the 25 biomarkers identified in the model group, it was found that RH can make 16 metabolic levels trend back to the control group. In RH group, the level of ornithine, 5’-Methylthioadenosine, hydroxytyrosol, prostaglandin F2a, phenylalanine, and arachidonic acid were decreased, and the level of isocitric acid, 3-hydroxyanthranilic acid, citric acid, asparagine, tryptophan, glutamine, SM (d18:1/22:0), cyclic GMP, taurocholic acid, LysoPC(17:0) were increased compared with CGN rats. Hierarchical clustering analysis was employed to describe the relationships and differences among metabolites and samples as shown in **Figure 4**. Metabolites related with RH medicinal effect in similar pathway or abundance patterns were sited closer together. Note that blood samples in the control and model groups were differentiated on the distinct branches, suggesting the CGN model was successfully established, the samples in the PA an RH group were clustered together near those in the control group, indicating that the content of the metabolites in the medicine treated and control groups was similar. Detailed comparisons of metabolite relative peak area in control, model, PA, and RH group are shown in **Supplementary Figure 2**.

### Metabolic Pathways Analysis

**Figure 5:**
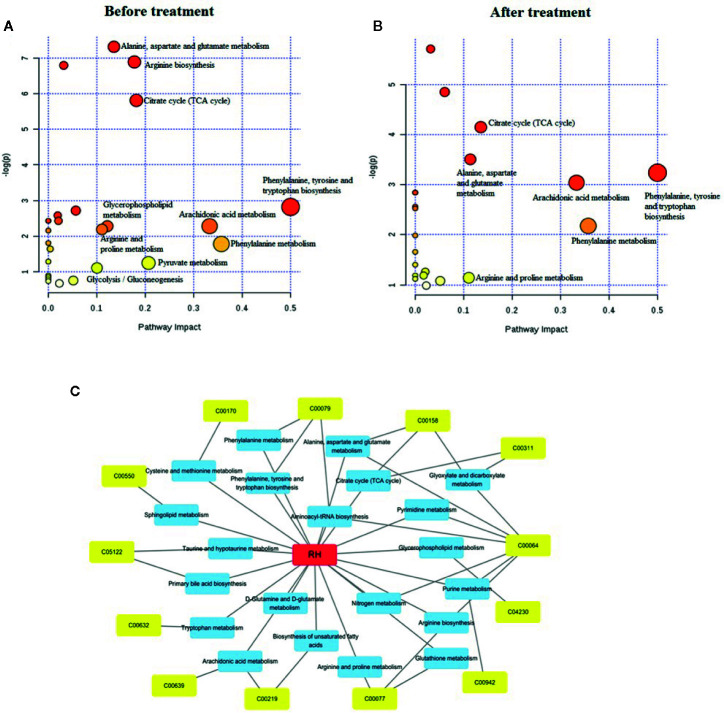
The altered metabolomic pathways associated with identified differential biomarker in serum sample before **(A)** and after RH treatment **(B)**, and the labeled pathways is p value > 0.1. A network diagram of compounds-metabolites-pathways is obtained from the MePtA online software **(C)**. The red color represents RH compounds, the blue represents all metabolic pathways affected by RH treatment, and the yellow colors represent metabolites closely related to the pathway.

From Pathway Analysis Features in MetaboAnalyst 4.0, CGN pathological changes are related to 25 differential metabolites, involving 20 metabolic pathways. Among them, the metabolic pathways with p value <0.1 including phenylalanine, tyrosine and tryptophan biosynthesis, phenylalanine metabolism, arachidonic acid metabolism, pyruvate metabolism, citrate cycle (TCA cycle), arginine biosynthesis, alanine, aspartate, and glutamate metabolism, glycerophospholipid metabolism, arginine and proline metabolism, glycolysis/gluconeogenesis. After RH treatment, the levels of 16 metabolites were regulated by RH, involving 21 metabolic pathways. Among them, the metabolic pathways with p value <0.1 including phenylalanine, tyrosine and tryptophan biosynthesis, phenylalanine metabolism, arachidonic acid metabolism, citrate cycle (TCA cycle), alanine, aspartate, and glutamate metabolism, arginine and proline metabolism (**Figures 5A, B**). The relationship between RH, corresponding differential metabolites and pathways related to RH treatment in CGN rats was exhibited in **Figure 5C**. Detailed the KEGG diagram of vital six pathways associated with RH protecting against CGN in rats were showed in **Supplementary Figure 3**. The information of single nucleotide polymorphisms (SNPs) loci and dysfunctional enzymes were detected by genome-scale network model of human metabolism in **Supplementary Figure 4**.

## Discussion

The prevalence of chronic kidney disease is increasing year by year and has become a global public health problem. In recent years, the technology of metabolomics has been widely used in the research of cardiovascular disease, liver disease, tumor, and other diseases, but the research of chronic kidney disease based on metabolomics technology is still relatively few (Zhang et al., 2015; Sun et al., 2016; Zhang Z. H. et al., 2016; Zhang Z. H. et al., 2018). Most of them focus on animal models, and there are few clinical studies. According to the pathway analysis, six metabolic pathways were related to the pharmacological effects of RH, and it was found that amino acid metabolism was the main factor role. Amino acids as an important class of active molecules in the organism can directly participate in the metabolism of the organism. Its catabolism is closely associated with the abnormal occurrence of protein biosynthesis, inflammation, and other diseases. By analyzing the combined database and mass spectrometry information, 16 related biomarkers were finally determined comparing the intrinsic differential metabolites between the control, model group, PA, and RH group. In light of the changing trend in blood samples and routine pharmacodynamic index testing, it was found that the differential metabolites refer to inflammatory response, oxidative stress, renal self-protection mechanism, immune system response, and glomerular filtration function.

Isocitric acid is an isomer of citric acid. In the TCA cycle, citric acid reversibly produces isocitric acid under the action of aconitase. Some studies were reported that isocitric acid and citric acid is related to diabetic nephropathy and chronic glomerulonephritis. Iron citrate is a drug that is effective in critically ill patients and is used to control serum phosphorus levels in patients with chronic kidney disease (CKD) dialysis (Hallan et al., 2017; Zhang Y. et al., 2018). Ornithine can be used for nutritional supplementation, acute and chronic liver diseases such as cirrhosis, fatty liver, hyperammonia caused by hepatitis and central nervous system symptoms. The ornithine cycle converts the more toxic ammonia produced by protein metabolism in the body into the less toxic urea, which is excreted from the body. Arginine is the precursor of ornithine and proline, which proline is an important element constituting collagen, and arginine supplementation play a vital role in severe tissue repair (Torres Montaguth et al., 2019; Li H. et al., 2019). 5’-methylthioadenosine (MTA) is a nucleoside produced from s-adenosylmethionine (AdoMet) during polyamine synthesis. Recent evidence suggests that AdoMet can regulate inflammatory mediators in the body. The effects of MTA are accompanied by inhibition of circulating tumor necrosis factor-α (TNF-α), inducible nitric oxide synthase (iNOS) expression, and stimulation of IL-10 synthesis. MTA can inhibit the transcriptional activation of iNOS by proinflammatory cytokines in liver cells, inhibit the induction of COX2 in raw264.7 cells, inhibit p38 mitogen-activated protein kinase (MAPK), c-jun phosphorylation, inhibitor kappa B alpha (IB) degradation, and nuclear factor B (NFĸB) activation, all of which are signaling pathways related to the production of inflammatory mediators (Eloranta et al., 1982; Li Y. et al., 2019).

3-Hydroxyanthranilic acid (3-HAA) is a tryptophan metabolite with anti-inflammatory activity, which the immunoregulatory molecular mechanism of 3-HAA on macrophages is inhibiting the production of inflammatory mediators and reducing NF-κB activity. The results show that 3-HAA has an immunomodulatory effect, which may be due to the inhibition of PI3K/Akt/mTOR and NF-κB activation, thereby reducing the production of proinflammatory mediators (Krause et al., 2011). Hydroxytyrosol can restrain NF-κB signal and lower LPS level, the expression of iNOS, cyclooxygenase-2, TNF-α and interleukin-1β is reduced, leading to the production of NO and prostaglandin E2 to exert anti-inflammatory effect (Zhang et al., 2012b; Fuccelli et al., 2018). Cyclic GMP as an important inhibitor of renal fibrosis synthesized by guanylate cyclase stimulated by nitric oxide or natriuretic peptide, and has pleiotropic regulatory functions in the kidney. The integration of cGMP-dependent protein kinases into cGMP signals may play an important role in the physiological processes of the kidney through cGKIα (Jankowski et al., 2001; Lieb et al., 2009). Arachidonic acid acts a critical role as a phospholipid-bound structural lipid in the blood, liver, muscle, and other organ systems. It is a biologically active substance of many circulating eicosanoid derivatives, such as prostaglandin E2 (PGE2) as well as prostacyclin (PGI2). Studies have reported that arachidonic acid can inhibit glomerular synthesis of thromboxane B-2, leukotriene B-4, and 12-hydroxyeicosatetraenoic acid, and alleviate glomerular filtration rate and renal blood flow. Urinary fluid loss (HP) and volume expansion (VE) were performed in patients with active IgA glomerulonephritis (IgA GN) for prostaglandin F2a. Urinary excretions of PGF2 and 6-keto-pgf1 level were significantly increased in patients with low glomerular filtration rate, suggesting that these substances play a role in advanced renal disease (Liang et al., 2016c; Wang et al., 2019; Wang et al., 2020).

Phenylalamine (PA) as an essential amino acid in the human body is involved in the formation of various protein components, but cannot be synthesized in the human body. Under normal circumstances, about 50% of the PA consumed is used to synthesize proteins of various components, and the rest is converted to tyrosine under the action of phenylalanine hydroxylase, and then converted into dopamine, epinephrine, norepinephrine, and melanin. When PA hydroxylase is lacking, these metabolites reach abnormally high levels and accumulate in tissues, plasma, and cerebrospinal fluid, which are excreted in large quantities from the urine (Alkaitis and Ackerman, 2016). Taurocholic acid can reduce capillary permeability of inflammatory tissues, inhibit inflammatory swelling, and restrain the production of inflammatory mediators such as NO, PGE2, and histamine. Asparagine is a drug used in clinical practice for lowering blood pressure, dilating bronchus (asthma), anti-peptic ulcer, and gastric dysfunction. Studies have found that elevated inflammatory mediators may increase kynurenine and tryptophan levels, leading to depression in patients with end stage renal disease (Zhang A. H. et al., 2014; Liang et al., 2016d; Liu Z. et al., 2018; Chiu et al., 2019). Glutamine can contribute to muscle growth and protein synthesis in muscle cells in the body. In addition, it enhances the function of the immune system, which is involved in the synthesis of glutathione as an important antioxidant. Glutamine can improves the body’s metabolic nitrogen balance, promotes protein synthesis, increases the total number of lymphocytes, reduces the release of inflammatory mediators and the body’s stress response (Vittorelli et al., 2005; Liang et al., 2016e). It was reported in the literature that phospholipid metabolism and sphingomyelin metabolism abnormalities are involved in the pathological development of diabetic nephropathy and advanced renal cancer. The levels of SM (d18:1/22:0), LysoPC (17:0), LysoPC (15:0), and PE (15:0/20:1) were decreased in this study, indicating that phospholipid metabolism and sphingomyelin metabolism are abnormal (Biernacki et al., 2018). With the continuous development of metabolomics technology and further research, its application in the field of disease is bound to be more extensive. It can be used to assist in the diagnosis and differential diagnosis of the disease, but also it can dynamically observe the progress of the disease, evaluate the clinical course of the disease (Liang et al., 2015b; Liang et al., 2016f; Zhang et al., 2017). In the future, better integration of metabolomics with genomics, transcriptomics, and proteomics to explain the biological significance of metabolic markers related to chronic kidney disease will be an urgent problem (Wang et al., 2014; Pinu et al., 2019).

## Conclusion

In this study, a LC/MS-based serum metabolomics method has been employed to explore the metabolic changes of CGN rats in response to RH treatment for fully insight into the anti-inflammatory activity of RH and its action mechanism in molecule level. Six several metabolism pathways including Hphenylalanine, tyrosine and tryptophan biosynthesis, phenylalanine metabolism, arachidonic acid metabolism, citrate cycle (TCA cycle), alanine, aspartate and glutamate metabolism, arginine and proline metabolism were regulated after RH treatment, which 16 metabolites were involved such as isocitric acid, ornithine, 5’-methylthioadenosine, 3-hydroxyanthranilic acid, as well as citric acid. The action mechanisms of RH against CGN were may be through the regulation of inflammatory response, oxidative stress, kidney protective function, and the immune system response to achieve.

## Data Availability Statement

The raw data supporting the conclusions of this article will be made available by the authors, without undue reservation, to any qualified researcher.

## Ethics Statement

The animal study was reviewed and approved by Animal Care and Use Committee of Harbin Medical University.

## Author Contributions

M-YZ conceived and designed the experiments. WYu, X-LM, WYa, and M-YZ performed the experiment. WYu and X-LM analyzed the data. WYu wrote the paper. All authors contributed to the article and approved the submitted version.

## Funding

This study was supported by grants from the National Natural Scientific Foundation of China (NO. 81772045), the Foundation of the First Affiliated Hospital of Harbin Medical University (NO.2017L004), and the Scientific Research Foundation of Heilongjiang Provincial Health Department (NO.2017-049).

## Conflict of Interest

The authors declare that the research was conducted in the absence of any commercial or financial relationships that could be construed as a potential conflict of interest.
