# Supplementary material for: Functional Metabolomics Analysis Elucidating the Metabolic Biomarker and Key Pathway Change Associated With the Chronic Glomerulonephritis and Revealing Action Mechanism of Rhein
Source: Front Pharmacol. 2020 Sep 25;11:554783. doi: 10.3389/fphar.2020.554783 (PMC7544993; doi:10.3389/fphar.2020.554783)
Supplement: Supplementary file 1 [file DataSheet_1.docx]

**Table S1** Differentially expressed metabolites in CGN rat using high-throughput metabolomics analysis after RH treatment.

| **No.** | **Mode** | **RT** | **Name** | **Chemical formula** | **m/z** | **HMDB code** | **VIP value** | **Trend in model** | **RH regulation** | **Statistical significance** |
| --- | --- | --- | --- | --- | --- | --- | --- | --- | --- | --- |
| 1 | M-H | 0.45 | isocitric acid | C6H8O7 | 191.02 | HMDB00193 | 2.3342 | ↓ | √ | ****** |
| 2 | M-H | 0.69 | Ornithine | C5H12N2O2 | 131.08 | HMDB00214 | 3.2132 | ↑ | √ | ****** |
| 3 | M+H | 0.99 | 5'-Methylthioadenosine | C11H15N5O3S | 298.09 | HMDB0001173 | 2.0717 | ↑ | √ | ****** |
| 4 | M+H | 1.27 | 3-Hydroxyanthranilic acid | C7H7NO3 | 171.08 | HMDB0001476 | 1.1790 | ↓ | √ | ****** |
| 5 | M+H | 1.56 | Citric acid | C6H8O7 | 215.02 | HMDB0000094 | 1.2978 | ↓ | √ | ****** |
| 6 | M-H | 1.88 | Argininosuccinicacid | C10H18N4O6 | 335.12 | HMDB0000052 | 2.1879 | ↑ |  |  |
| 7 | M+H | 2.17 | Uric acid | C5H4N4O3 | 169.04 | HMDB00289 | 2.4052 | ↑ |  |  |
| 8 | M-H | 2.56 | Asparagine | C4H8N2O3 | 131.05 | HMDB33780 | 1.3987 | ↓ | √ | ****** |
| 9 | M+H | 3.27 | Tryptophan | C11H12N2O2 | 205.10 | HMDB13609 | 1.1123 | ↓ | √ | ***** |
| 10 | M-H | 3.78 | Glutamine | C5H10N2O3 | 145.06 | HMDB00641 | 2.3434 | ↓ | √ | ****** |
| 11 | M-H | 4.03 | SM(d18:1/22:0) | C45H91N2O6P | 785.65 | HMDB12103 | 1.4432 | ↓ | √ | ***** |
| 12 | M-H | 4.49 | Cervonoylethanolamide | C24H36O3 | 373.27 | HMDB13627 | 1.8760 | ↓ |  |  |
| 13 | M-H | 4.77 | Cysteinylglycine | C5H10N2O3S | 179.05 | HMDB00078 | 2.7342 | ↓ |  |  |
| 14 | M-H | 5.21 | Hydroxytyrosol | C8H10O3 | 153.06 | HMDB0005784 | 4.8948 | ↑ | √ | ***** |
| 15 | M+H | 5.62 | Cyclic GMP | C10H12N5O7P | 368.04 | HMDB0001314 | 3.3718 | ↓ | √ | ****** |
| 16 | M-H | 5.88 | Prostaglandin F2a | C20H34O5 | 399.24 | HMDB0001139 | 1.4901 | ↑ | √ | ****** |
| 17 | M-H | 6.29 | Taurocholic acid | C26H45NO7S | 514.28 | HMDB00036 | 3.2343 | ↓ | √ | ****** |
| 18 | M-H | 6.45 | Pyruvic acid | C3H4O3 | 87.01 | HMDB00243 | 3.0989 | ↓ |  |  |
| 19 | M+H | 6.71 | Phenylalanine | C9H11NO2 | 166.08 | HMDB00159 | 2.6575 | ↑ | √ | ****** |
| 20 | M+H | 6.86 | Arachidonic acid | C20H32O2 | 327.23 | HMDB01043 | 4.0321 | ↑ | √ | ****** |
| 21 | M-H | 7.42 | LysoPC(17:0) | C25H52NO7P | 508.34 | HMDB12108 | 1.5464 | ↓ | √ | ***** |
| 22 | M+H | 8.19 | LysoPC(15:0) | C23H48NO7P | 482.32 | HMDB10381 | 1.9888 | ↓ |  |  |
| 23 | M-H | 8.62 | Palmitoleic acid | C16H30O2 | 253.22 | HMDB03229 | 2.0998 | ↑ |  |  |
| 24 | M+H | 9.30 | Salbutamol | C13H21NO3 | 240.16 | HMDB0001937 | 1.3962 | ↑ |  |  |
| 25 | M+H | 9.66 | PE(15:0/20:1) | C40H78NO8P | 732.56 | HMDB08900 | 2.5407 | ↓ |  |  |

Note : “*” , p<0.05; “**”, p<0.01.

Compared with control group, “↓”stands for the decreasing level and “↑”stands for the increasing level.

“√” stands for differentiated metabolites that were regulated by RH treatment


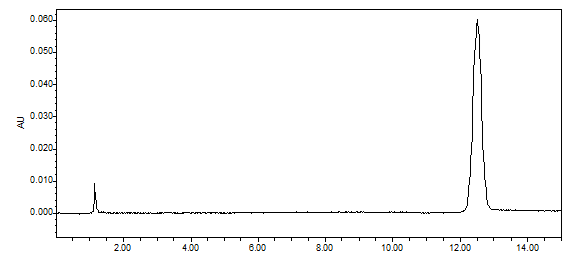


Supplementary figure 1. The chromatogram of rhein detected using HPLC. Agilent XDB-C18 column (250mm×4. 6mm, 5μm) was performed; the mobile phase was acetonitrile-methanol-0.1% phosphoric acid solution (39:20:41); the volume flow rate was 1.0mL • min-1; the column temperature was 30 ℃; the detection wavelength is 258 nm.


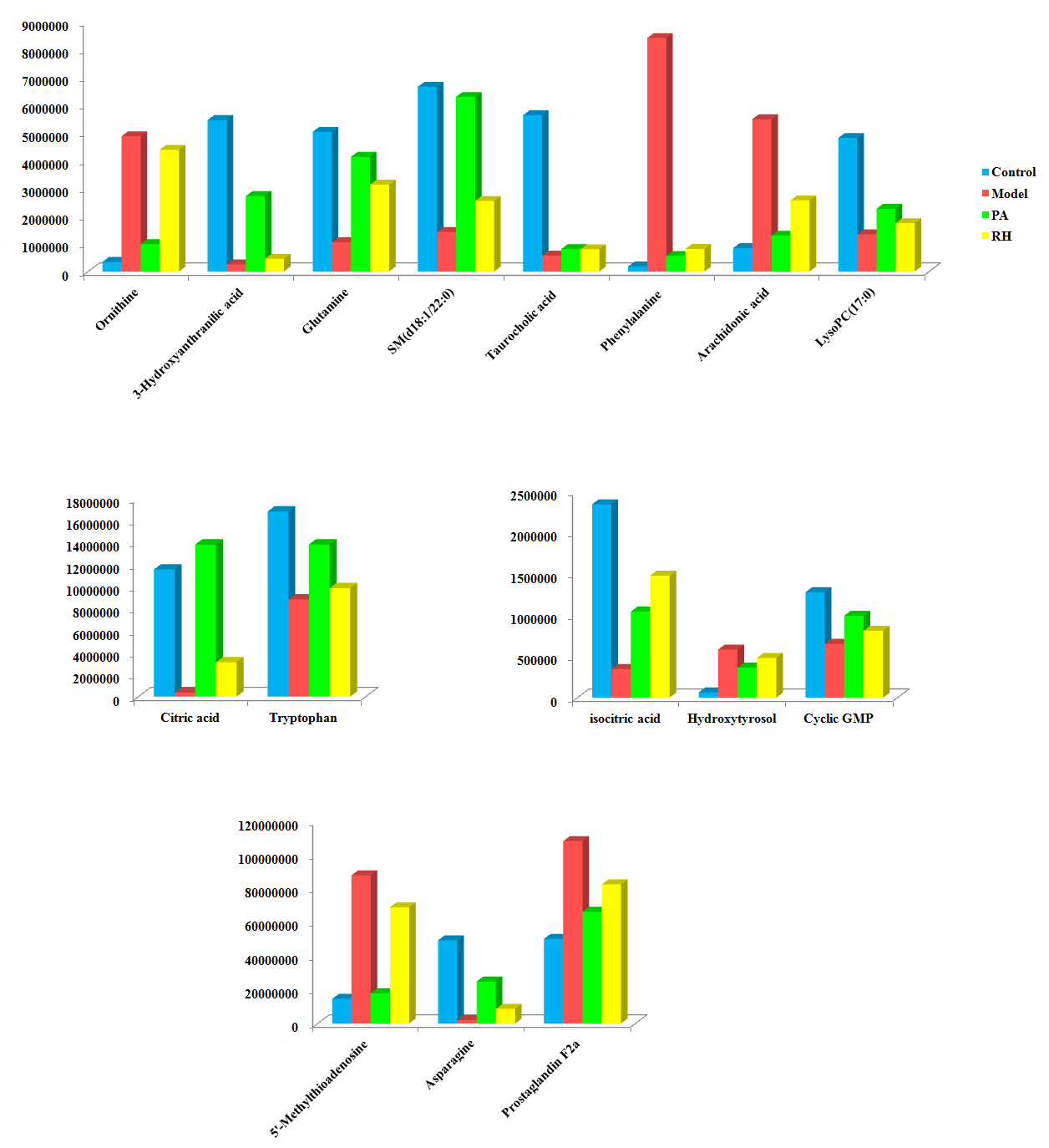


Supplementary figure 2. Relative signal intensities of serum metabolites in different groups identified by LC-MS, which the result of significant difference comparison between the groups are listed in Table S1.


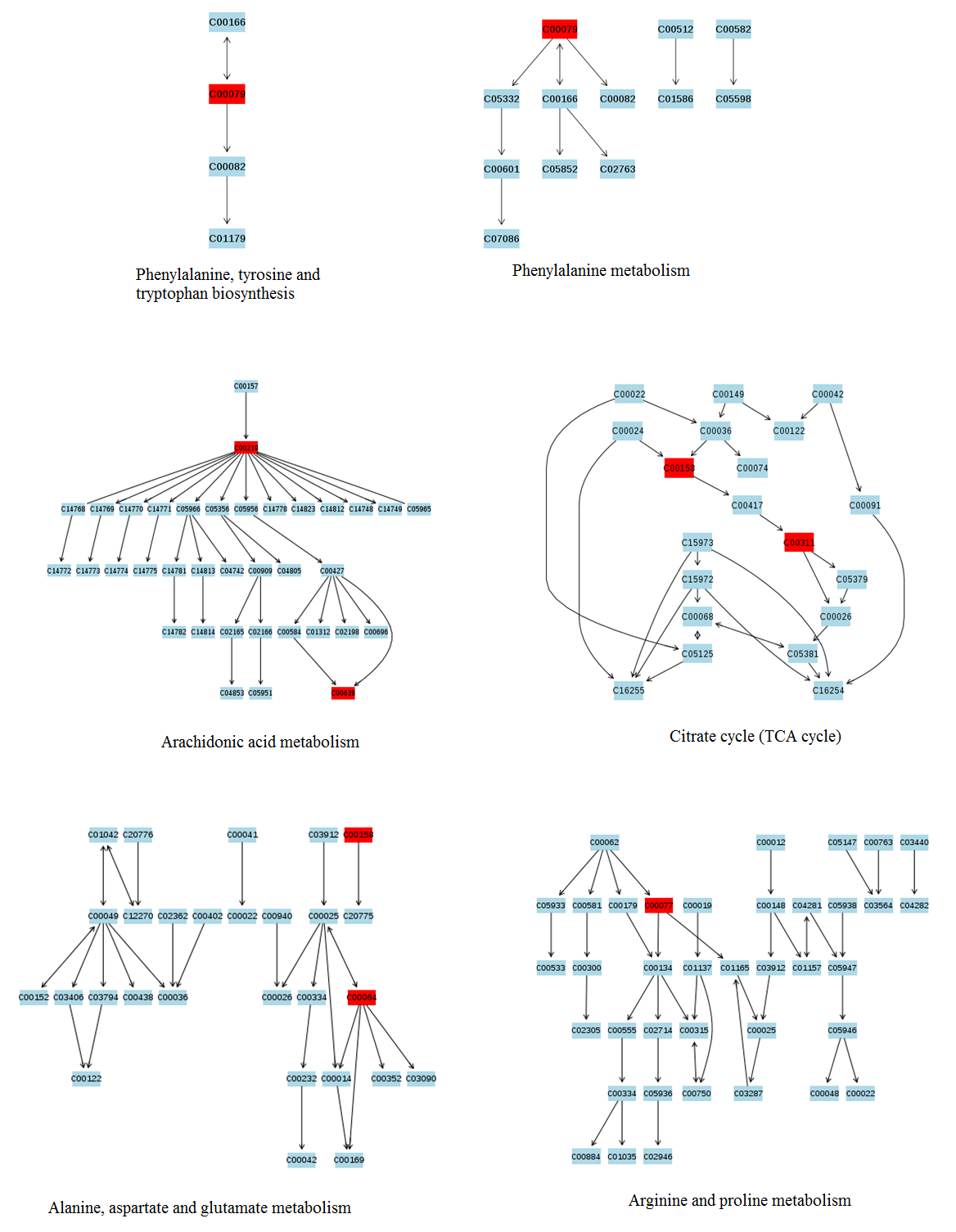


Supplementary figure 3. Detailed KEGG network correlation diagram for VIP values greater than 0.1 after RH treatment.


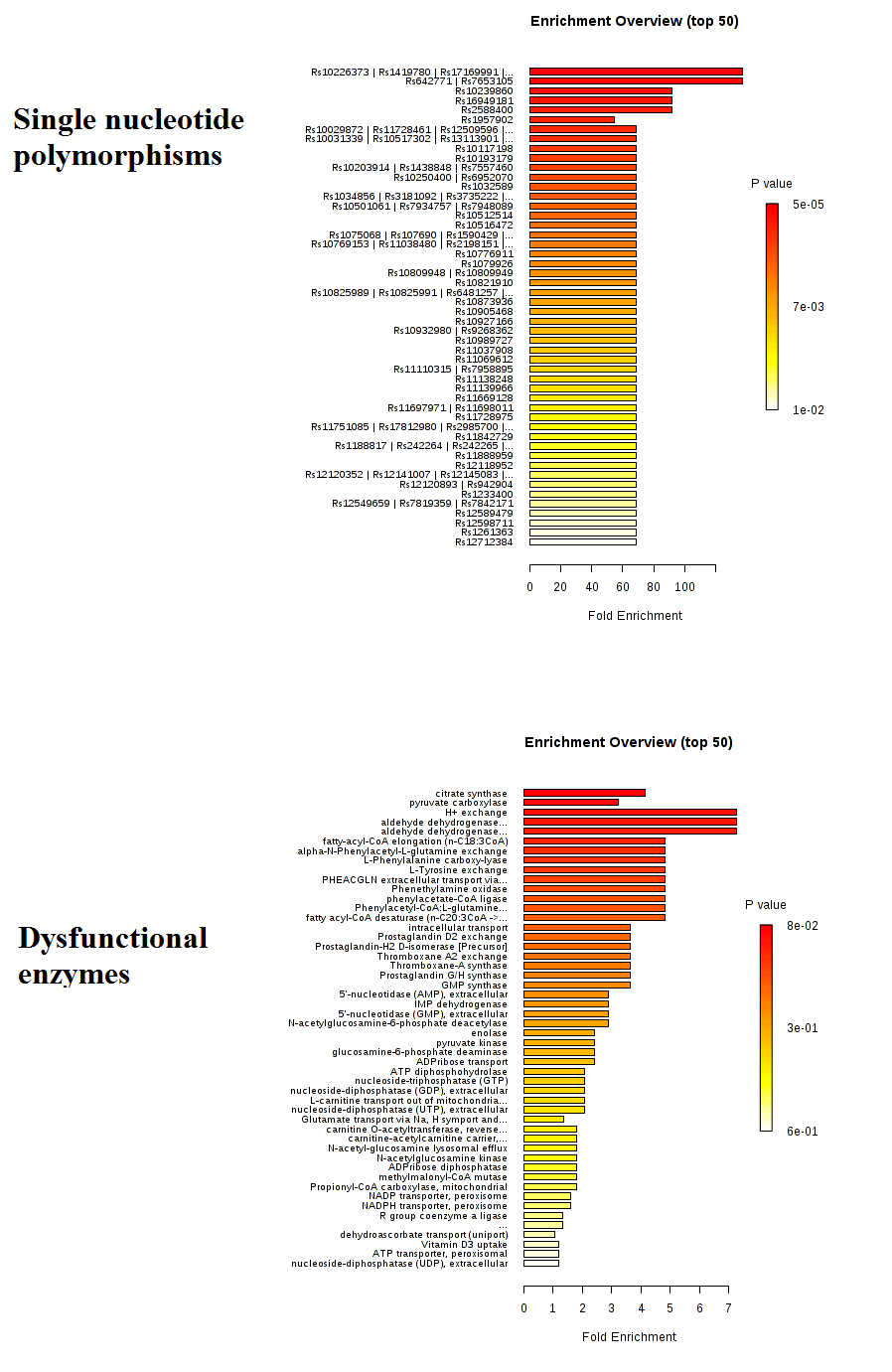


Supplementary figure 4. The information of single nucleotide polymorphisms (SNPs) loci and dysfunctional enzymes were detected by genome-scale network model of human metabolism after RH treatement on CGN rats.
